# Biodegradable Polycaprolactone-Titania Nanocomposites: Preparation, Characterization and Antimicrobial Properties

**DOI:** 10.3390/ijms14059249

**Published:** 2013-04-29

**Authors:** Alexandra Muñoz-Bonilla, María L. Cerrada, Marta Fernández-García, Anna Kubacka, Manuel Ferrer, Marcos Fernández-García

**Affiliations:** 1 Instituto de Ciencia y Tecnología de Polímeros (ICTP-CSIC), C/Juan de la Cierva 3, 28006 Madrid, Spain; E-Mails: mlcerrada@ictp.csic.es (M.L.C.); martafg@ictp.csic.es (M.F.-G.); 2 Instituto de Catálisis y Petroleoquímica, (ICP-CSIC), C/Marie Curie 2, 28049 Madrid, Spain; E-Mails: mferrer@icp.csic.es (M.F.); mfg@icp.csic.es (M.F.-G.)

**Keywords:** titania, polycaprolactone, nanocomposites, thermal properties, antimicrobial

## Abstract

Nanocomposites obtained from the incorporation of synthesized TiO_2_ nanoparticles (≈10 nm average primary particle size) in different amounts, ranging from 0.5 to 5 wt.%, into a biodegradable polycaprolactone matrix are achieved via a straightforward and commercial melting processing. The resulting nanocomposites have been structurally and thermally characterized by transmission electron microscopy (TEM), wide/small angle X-ray diffraction (WAXS/SAXS, respectively) and differential scanning calorimetry (DSC). TEM evaluation provides evidence of an excellent nanometric dispersion of the oxide component in the polymeric matrix, with aggregates having an average size well below 100 nm. Presence of these TiO_2_ nanoparticles induces a nucleant effect during polymer crystallization. Moreover, the antimicrobial activity of nanocomposites has been tested using both UV and visible light against Gram-negative *Escherichia coli* bacteria and Gram-positive *Staphylococcus aureus*. The bactericidal behavior has been explained through the analysis of the material optical properties, with a key role played by the creation of new electronic states within the polymer-based nanocomposites.

## 1. Introduction

Food packaging plays a decisive role in achieving protection and preservation of all types of food, particularly from oxidative and microbial spoilage, as well as dehydration and, therefore, extends the shelf life of the food product. Synthetic and non-biodegradable materials are most often used for food packaging and accumulation of which poses a threat to the environment. The most effective solutions to promote both food and environment preservation, in this type of packaging might comprise, on one hand, the replacement of such materials for other environmentally friendly packaging ones based, for instance, on biodegradable polymers, and, on the other hand, the incorporation of some antimicrobial agent that minimizes and even prevents the growth and adhesion of detrimental microorganisms. Concerning the former aspect, poly(ɛ-caprolactone), PCL, which is a semicrystalline linear aliphatic polyester, could be a good candidate. Additional characteristics that can be mentioned include its flexibility, non-toxicity, hydrophobicity and ease to be processed [1–3]. Related to the second point, titania might be an excellent alternative. TiO_2_ is an inert and cheap material, and its well-known non-toxicity (even in nanometric scale) for human related applications allows its use as an additive in cosmetics, pills or toothpaste [4]. Additionally, TiO_2_ is able to eliminate (within an extended period of time) dead cells, rendering CO_2_, opening, in this way, a path for the auto-regeneration of the system [5–7]. A point of relevance is the control of the TiO_2_ polymorphism ensuring the presence of the anatase form, the one with the highest biocidal capability, as well as to control primary particle size in the nanometer range, a fact that would limit scattering events among other things [8,9]. TiO_2_-anatase works under UV light excitation with energy above the corresponding band gap (*ca*. 3.2 eV), forming energy-rich electron-hole pairs. Such charge carriers are able to interact with microorganisms, rendering biocidal properties to the corresponding polymer-based nanocomposite films [10–13].

Organo-inorganic nanocomposite materials that combine attractive qualities of dissimilar oxide and polymer components are not simply physical blends, but they can be broadly defined as complex materials containing both organic and inorganic constituents intimately mixed. The scale of mixing or, in other words, the degree of homogeneity, would influence or even command the ultimate properties of the solid nanocomposite materials when the component mixture is adequately reached, typically at the nanometer range [14,15]. In particular, the optimization of the component contact has been shown to be crucial in order to render TiO_2_-containing polymer nanocomposites with outstanding biocidal properties [10–13,16,17]. All these together features might lead to a safer, cost-effective technology of general application as an alternative to current inorganic- and organic-based biocidal agents.

The goal of this work is, then, to gain knowledge on the universality of using TiO_2_-anatase nanoparticles to impart antimicrobial characteristics in polymer-based nanocomposites. An efficient organo-inorganic contact has been previously proved to change the nature of the TiO_2_ agent, avoiding the requirement of a close proximity with the pathogen and making the oxide in the nanocomposite a non-contact agent [17]. Then, the preparation and complete characterization of polycaprolactone-TiO_2_ nanocomposites are here reported. Several contents of the inorganic component have been evaluated, and a comprehensive structural characterization of the different organic-inorganic nanocomposites has been performed because of the feasible effect of TiO_2_ on the polymeric structural details (crystallinity, crystal size, melting and crystallization temperatures) and, consequently, on the overall physical properties. Therefore, through a multi-technique approach, the study aims to show that adequate incorporation of TiO_2_ into a biodegradable PCL leads to a powerful antimicrobial system, with a biocidal potential higher than that of the oxide alone.

## 2. Results and Discussion

**Figure 1:**
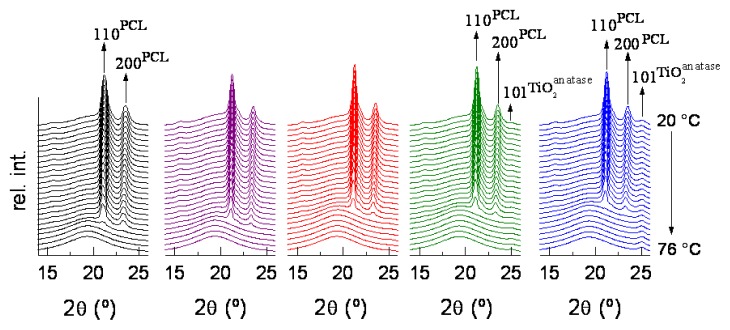
Real-time variable-temperature wide angle X-ray diffraction (WAXS) profiles obtained with synchrotron radiation for all the samples in a melting experiment at 8 °C/min. From left to right: PCL-TiO_2_-0; PCL-TiO_2_-05; PCL-TiO_2_-1; PCL-TiO_2_-2 and PCL-TiO_2_-5. PCL, poly(ɛ-caprolactone).

The polycaprolactone is semicrystalline and wide angle X-ray diffraction (WAXS) experiments were performed to determine the influence of TiO_2_ nanoparticle incorporation on the crystal lattice developed in the different nanocomposites. Similar WAXS profiles are found from room temperature to the molten state for all the hybrid samples and the PCL polymeric component, as depicted in Figure 1. Therefore, all of these quenched specimens show an orthorhombic lattice, without noticeable changes with respect to the profile exhibited by the neat PCL. In addition to the rather constant 110 and 200 diffraction peaks characteristic of the PCL orthorhombic cell [18], the 101 reflection ascribed to the TiO_2_ anatase polymorph (centered at *ca*. 25°; JCPDS-84-1286) is seen in the nanocomposites and its intensity increases as the TiO_2_ content does. The crystallinity degree of the different nanocomposites can be estimated from these WAXS patterns by their decomposition into crystalline diffractions and the amorphous component, since the amorphous halo is not known at a given temperature (it is only characterized in the molten state). The amorphous peak of the different samples was found at room temperature to be centered at 2θ = 19.8 ± 0.2 after deconvolution. Its position can be undoubtedly determined after the melting process has taken place. Values are listed in Table 1 and show that position of the most probable intermolecular distance between PCL macromolecules (*d*^HALO^) is slightly moved to higher spacing values with increasing TiO_2_ content. Variation is subtle, but seems to indicate that favorable PCL-TiO_2_ interactions are being created, at least, in the molten state, and these interactions weaken those existing between polymeric macromolecules. Table 1 also reports the crystallinity results calculated at room temperature. It is clearly observed that this magnitude remains nearly unchanged by the presence of TiO_2_ nanoparticles.

**Figure 2:**
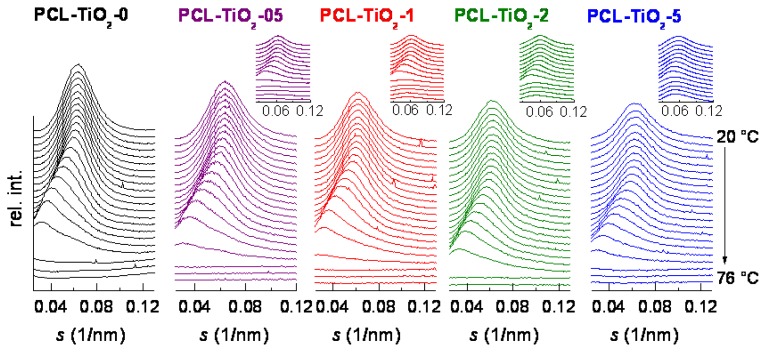
Real-time variable-temperature Lorentz-corrected SAXS profiles (main plots: only the polymeric contribution; insets: initial patterns in the nanocomposites: TiO_2_ and polymeric contributions) obtained with synchrotron radiation for all the samples as processed in a melting experiment from 20 to 76 °C at 8 °C/min.

Real time variable-temperature SAXS experiments were also performed to estimate long spacing values and the effect of TiO_2_ incorporation. PCL-TiO_2_-0 profiles, as well as those represented in the distinct insets of Figure 2 show the initial profiles during heating for all of the samples analyzed. Several characteristics are common independently of TiO_2_ content: (a) an important shift of the peak to lower *s* values (*i.e*., a rise of long spacing, *L*SAXS, since *s* = 1/*d* and *d* = *L*SAXS) with increasing temperature and this fact indicating changes in the PCL lamellar thickness, in its amorphous layer thickness or in both of them; (b) the existence of a periodicity peak after melting of the polymeric matrix in all the nanocomposites (see the insets in Figure 2 for the distinct hybrids). This correlation peak is ascribed to a characteristic spacing between TiO_2_ nanoparticles [19]. Therefore, two specific contributions should be initially taken into consideration: the one associated with differences in electronic density between crystallites and amorphous regions in the PCL component and that regarding the presence of TiO_2_ in the resulting materials.

To exclusively evaluate the characteristics of the PCL crystallites, the latest contribution has been subtracted, as depicted in the patterns represented in Figure 2. Then, values of the most probable long spacing can be deduced from these Lorentz-corrected SAXS profiles [20–22] and those found at room temperature are reported in Table 1. A slight increase of the long spacing is detected, a fact that seems to point out that slightly thicker PCL crystallites are developed in the nanocomposites with respect to those existing in the pristine PCL matrix. To confirm this statement, the most probable crystallite size in the direction normal to the lamellae, *l*_c_, has been determined by assuming a simple two-phase model, *i.e*., *l*_c_ = *L*^SAXS^ × *f_c_*^NORM^_WAXS_. The values attained are also listed in Table 1, showing very small differences between specimens.

**Figure 3:**
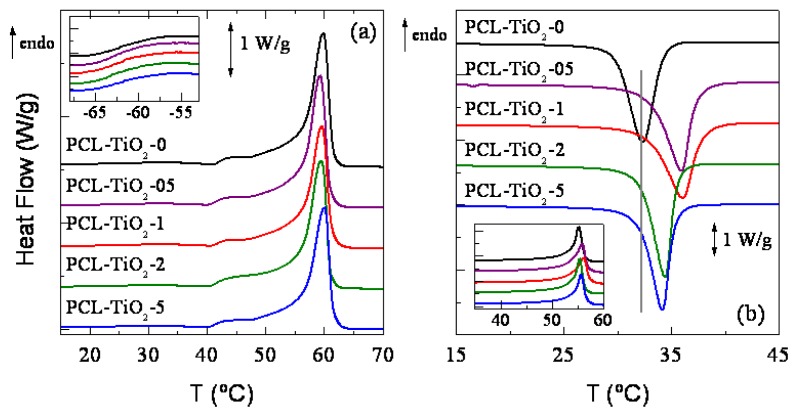
DSC curves corresponding to: (**a**) the first heating process and (**b**) the crystallization corresponding to the pristine PCL homopolymer and its nanocomposites with distinct TiO_2_ nanoparticle contents. The insets in (**a**) and (**b**) plots are related to the glass transition region and to the subsequent heating run after crystallization process, respectively.

The crystalline nature of these nanocomposites can be also evaluated from calorimetric measurements (see Figure 3a,b for first melting and crystallization processes, respectively). Two endothermic processes take place during the first heating run, the former one at very low temperatures ascribed to the glass transition (see inset in Figure 3a) and that related to the melting of crystallites at higher temperatures. Glass transition temperature, *T_g_*, remains rather constant independently of the TiO_2_ content (values reported in Table 2), a feature that indicates that mobility within the PCL amorphous regions does not vary much with TiO_2_ incorporation. On the other hand, the initial melting process shows multiple melting stages, since the crystalline phase transitions of PCL samples strongly depends on thermal history. Then, melting peaks from the DSC traces vary for samples stored at room temperature for different periods of time, and long-term maintained samples usually exhibit higher melting temperature. This fact implies that there is a progress of the crystalline structure at room temperature in the PCL-based samples, because of its low glass transition temperature, although it occurs at a rather slow pace. This feature is clearly deduced when comparing the first melting curves (Figure 3a) with those coming from the second heating run (inset in Figure 3b). Accordingly, melting temperature and crystallinity are, at a given specimen after a week at room temperature, higher than the ones attained just after crystallization, in spite of the cooling rate imposed in the initial film processing being much faster than the one applied along calorimetric measurement. This structural evolution makes it necessary to be very careful with these samples. Therefore, the PCL-based specimens must have an identical and well-defined thermal protocol (time elapsed from their processing and storage temperature) in order to obtain reproducible and reliable results.

This multiple melting behavior in polymers has been explained by assuming successive melting-recrystallization processes [23–26], existence of dual lamellar populations [27,28] or presence of two or more polymorphs [29–32]. The former assumption seems to be the cause for this thermal response in the specimens under study, since a unique PCL crystalline structure is developed, as deduced from WAXS measurements and two crystallite populations have not been apparently observed, as revealed in the SAXS profiles within the temperature range of interest. Consequently, melting of smallest and thinnest crystals takes place at low temperatures, and this molten material undergoes continuous recrystallization-melting phenomena with increasing temperature to form larger and thicker lamellae that melt at higher temperatures. Table 2 shows similar melting temperatures in the pristine PCL and the different nanocomposites, these results being in agreement with the rather analogous values of most probable crystal size deduced from combination of WAXS and SAXS measurements.

Enthalpy of melting can be also determined from these melting curves, and consequently, the DSC crystallinity can be estimated if the enthalpy of melting for the 100% crystal is previously known. A value of 135 J g^−1^ has been considered [33,34], and the results obtained after normalizing by the actual PCL amount at each hybrid are reported in Table 2. There is not considerable variation in the degree of crystallinity developed in the different samples, as already observed from the values calculated at room temperature from WAXS patterns. Nevertheless, the DSC values are greater than those determined from WAXS profiles, since part of the material in amorphous state at room temperature is able to crystallize during the heating processes and contribute to the final degree of ordering.

Figure 3b shows the PCL crystallization process for the different samples. It is clear the nucleating effect that TiO_2_ nanoparticles has on the PCL matrix. Consequently, crystallization temperatures, *T_c_*, are moved to higher values (see Table 2). The influence as nucleant that TiO_2_ exerts seems to be slightly dependent on the content, and thus, a less significant effect is observed as TiO_2_ incorporation is increased in the nanocomposite. This shift of *T_c_* is not related to a variation of crystallinity, but most probable crystallite size is, however, slightly enlarged as deduced from values estimated from SAXS and from the subsequent melting temperatures, *T_m_*^F2^.

**Figure 4:**
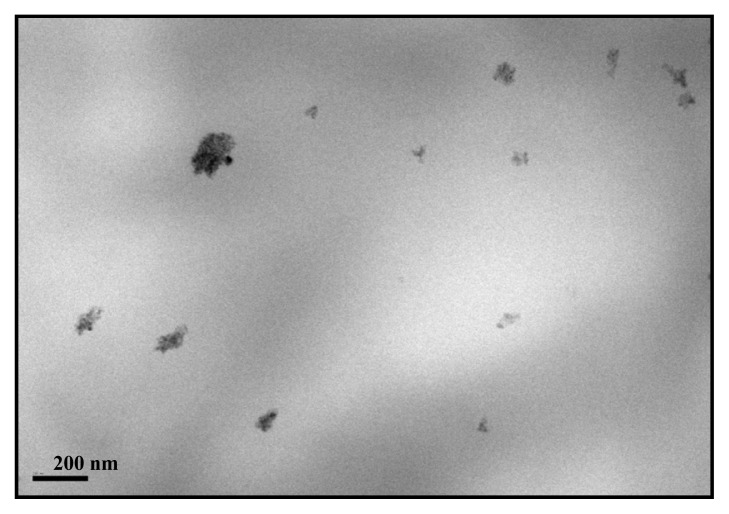
TEM micrographs of the PCL-TiO_2_-2 nanocomposite.

To gain knowledge on how inorganic component is arranged within the PCL matrix, because of its impact in the final performance of the resulting materials, TEM analysis was carried out. Figure 4 shows a uniform dispersion of inorganic nanoparticles within the polymer for the PCL-TiO_2_-2 nanocomposite and the non-existence of TiO_2_ aggregates of a large size. This behavior parallels the one observed in the other TiO_2_-based nanocomposite systems [12,13,16,17] prepared by melting processing. The oxide is dispersed in the polymeric matrix exhibiting nanometer-scale aggregates ranging from 10 (the oxide primary particle size) to 180 nm, with an average size (Feret diameter) [35] of *ca*. 80 nm. The nanometric dispersion of the oxide attained in the loadings analyzed up to 5 wt.% is significant, considering that the titania preparation makes use of an oxide previously calcined at high temperature to ensure the exclusive presence of the anatase polymorph and, then, a strict control of its biocidal properties.

**Figure 5:**
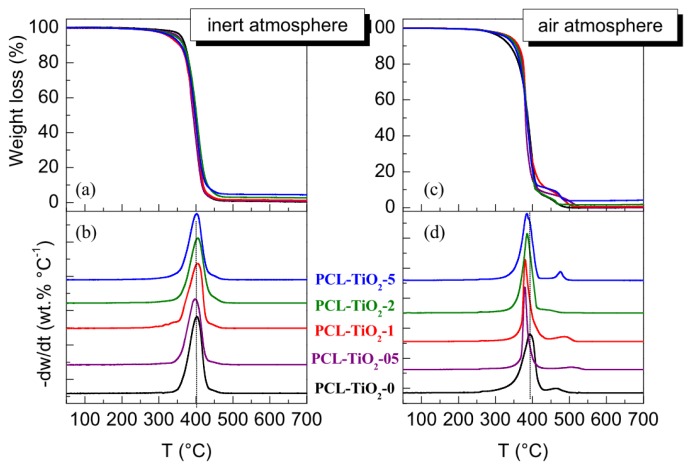
Left plot: (**a**) TG and (**b**) DTG curves obtained under an inert atmosphere; right plot: (**c**) TG and (**d**) DTG curves obtained under an oxidant atmosphere of pristine PCL homopolymer and the different nanocomposites analyzed. DTG curves have been vertically shifted for clarity.

Incorporation of well-dispersed nanoparticles within polymeric matrices has been proved to induce, sometimes, thermal degradation of the final materials. Accordingly, a very significant catalytic effect has been reported in hybrids based on Al nanoparticles within a poly(vinylidene fluoride) (PVDF) matrix [36], either under inert or oxidant atmospheres and an important shift of degradation temperatures, around 100 °C down to that found in the pristine matrix, even at a content as low as 1.5 wt.%. Mesoporous MCM-41 silica has promoted the thermal decomposition of hybrids based on polyethylene [37]. Nevertheless, incorporation of Cu nanoparticles into PVDF maintained and even slightly delayed the primary thermal decomposition in experiments performed under inert conditions [38]. Addition of TiO_2_ has displayed different effects depending on the polymeric matrix in which it is incorporated: a considerable catalytic effect in nanocomposites based in ethylene-vinyl alcohol (EVOH) copolymer or an almost null action in polypropylene [39,40]. Figure 5 shows the results related to the thermal stability of these PCL-based nanocomposites under inert and oxidant conditions. Looking first at curves achieved under a nitrogen atmosphere (Figure 5a), it can be said that degradability is nearly not affected by incorporation of oxide nanoparticles and, moreover, decomposition seems to take place in a unique stage (Figure 5b). Only, the onset temperature of decomposition is slightly moved to lower temperature. This picture is somehow different to that obtained when thermal stability is evaluated under an oxidant environment (Figure 5c). In this case, the degradation mechanism is more complex and takes place in several stages (Figure 5d). In addition, the presence of TiO_2_ nanoparticles slightly favors degradability, and the temperature of maximum degradation is shifted toward values lower than that corresponding to the PCL homopolymer. Nevertheless, the onset of degradation in the nanocomposites is moved to higher temperatures about 20 °C, this means that the beginning of the degradation process is a little postponed with the incorporation of TiO_2_ under air conditions. On the other hand, the TiO_2_ content can be estimated from the residual mass at the end of the experiments. A good agreement was found when comparing the averaged values with the theoretical loadings: 0.4%, 1.0%, 2.3% and 4.5% for the PCL-TiO_2_-05, PCL-TiO_2_-1, PCL-TiO_2_-2 and PCL-TiO_2_-5, respectively.

**Figure 6:**
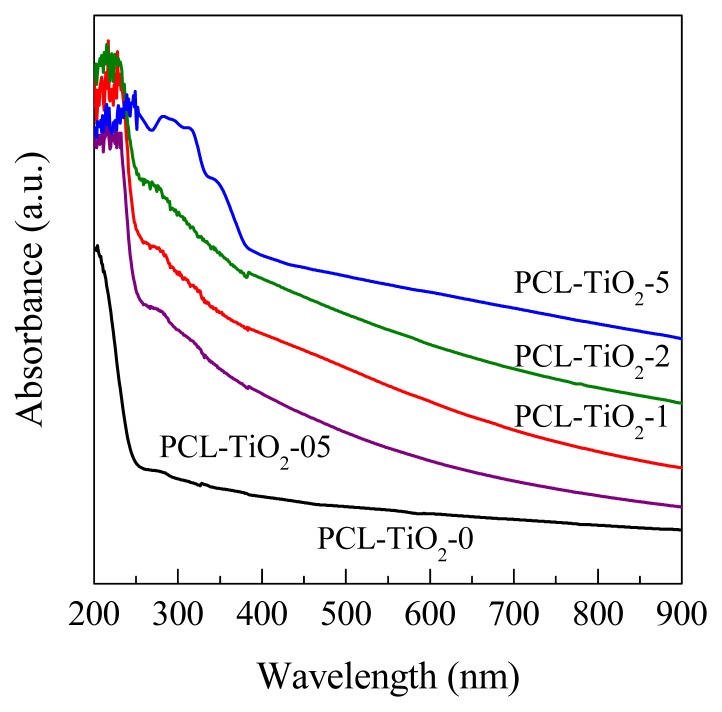
UV-visible absorption spectra of the PCL component and PCL-TiO_2_-*x* nanocomposite materials.

The presence of TiO_2_ in these nanocomposites has revealed non-significant changes in the PCL crystalline structure, phase transitions and thermal stability. Nevertheless, a more critical aspect is the knowledge of the effect of TiO_2_ on their optical properties, since they are rather important considering the need of efficient light absorption by TiO_2_ to become a biocidal material. The spectrum of the TiO_2_ component has been reported elsewhere [8,9,41] and is characterized by a band gap of 3.2 eV (*ca*. 380 nm). The UV-visible spectra of the nanocomposites and reference PCL are displayed in Figure 6. The PCL polymeric component shows a weak shoulder at 250–300 nm, which is attributed to the n→π* transition of the ester carbonyl. Incorporation of the inorganic component to the PCL polymeric matrix leads to important changes in the absorption profile. Inorganic nanoparticle loadings below 2 wt.% mainly alter the PCL spectrum by enhancing the absorbance on the visible region. Changes are more dramatic at 5 wt.% content and a new, broad absorption feature between 330 and 400 nm becomes evident. Therefore, presence of the inorganic component has two effects: enhancement of the signal intensity in the UV-A and visible ranges and indication of the characteristic oxide band gap. This last feature is only clearly distinguishable in the sample with the highest TiO_2_ content (PCL-TiO_2_-5). Light interaction with the nanocomposite materials would thus display a complex behavior, with an improvement of the absorption power of the nanocomposite that is not easily ascribed to any of the bare components, this being particularly true in the visible region because neither of them absorb light.

**Figure 7:**
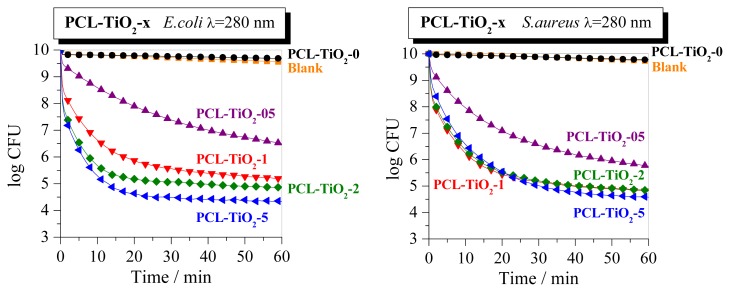
Survival curves after irradiation at 280 nm for two microorganisms (*E. coli* and *S. aureus*, top and bottom plots, respectively) as a function of the irradiation time for PCL-TiO_2_-*x* and PCL control samples.

Performance of the PCL reference, as a control sample, and the PCL-TiO_2_-05, PCL-TiO_2_-1, PCL-TiO_2_-2 and PCL-TiO_2_-5 films were tested to analyze the biocidal properties of these organic-inorganic systems. Experiments measuring the UV-alone effect on the microorganism survival ratios were also run. Results are depicted in Figure 7 for the two different microorganisms employed: the Gram-negative *Escherichia coli* (*E. coli*) and Gram-positive *Staphylococcus aureus* (*S. aureus*) bacteria. These measurements intend to establish the potential of these nanocomposite films against pathogens involved in the contamination and/or deterioration of packaged foods [5,42].

A first general point to stress out is that both control experiments (e.g., UV-alone and UV + PCL) produce only very moderate effects on the total log-reduction. The presence of TiO_2_ extremely affects the bio-killing action in the nanocomposite materials. A log-reduction ranging from 3 to 5.6 is observed, depending on TiO_2_ content at the end of the experiment against *E. coli* (e.g., 99.9% to 99.999% killing), whereas it ranges from 4 to 5 for *S. aureus*. This killing level is commonly understood as being bactericidal and, as detailed elsewhere, is sufficient to maintain a good (human health) safety control, helping in eliminating the need for sterilization or other aggressive treatments of foods [43]. Concerning the optimum TiO_2_ loading within the nanocomposites, non-definitive conclusion can be reached, since Figure 7 provides evidence of a distinct trend observed after UV irradiation, depending on the microorganism analyzed. Then, an increasing log-reduction is seen for *E. coli* as TiO_2_ is raised in the nanocomposite, but PCL-TiO_2_-1, PCL-TiO_2_-2 and PCL-TiO_2_-5 hybrids exhibit a similar biocidal behavior against *S. aureus*, this being better than that shown by the PCL-TiO_2_-05 specimen. The maximization of the bio-killing action is for *E. coli* somehow parallel to the morphological aspects (*i.e*., good dispersions and non-existence of agglomerates of large size) and the optical properties, both being relied on the correct handling of the matter-light interaction. It seems that an efficient charge carrier separation has been achieved upon the mixing of the polymer, and oxide components for samples having TiO_2_ content above 1 wt.% are of particular importance. Holes should be preferentially located in the organic component after charge carrier separation [17], being thus able to interact with microorganisms without the need of a direct oxide-biological contact.

Comparison with previous results concerning the efficiency of inorganic/organic biocidal agents is easy in the case of the *E. coli* [44–50] and *S. Aureus* [51,52] microorganisms. There is also an important number of works devoted to both pathogens [53–59]. To make appropriate comparison of biocidal properties, we must consider at least three points: the first one relates to the rather different excitation energies and/or fluences (power × time) used in experiments with titania, as both influence significantly the results. The second concerns the state and nature of the biocidal agent; while certain biocidal agents, as Ag, are typically released to the media, others, like TiO_2_, works by surface/near-surface contact and displays significant variability in efficiency while used as powders or immobilized in a support. As is well known, as a powder, it may present typically a one-fold order of magnitude higher activity (for example, in the case of *E. coli*) [60], due to the fact that nanoparticles in suspension/powders can be ingested by microorganisms via phagocytosis, causing rapid cellular damage in addition to that caused by photo-activity [44]. The third point is specific for the measurements of cell inactivation reaction rates, as they show a linear relationship with the initial bacteria concentration, a fact that must be taken into account when comparing results [61].

*E. coli* control and elimination have received a lot of attention as a general model system, with reports using TiO_2_ powders [44–48,53], immobilized/supported TiO_2_ on polymers (acetate; polystyrene, Plexiglas) [49,54,55,60] or Ag [50,57,58] biocidal agents. Considering that our films contain a “immobilized” TiO_2_ oxide, the maximum log-reduction (5.6) obtained after 1 h of treatment further confirms the goodness of the nanocomposite films. Measurements using immobilized/supported TiO_2_ systems give values of *ca*. 5.4/2/1 log-reduction for 1.5/0.5/1 h of treatment [49,54,55]. To end, the comparison can be extended to Ag-based biocidal agents; while a general higher activity is observed for our system, we can make a direct, useful comparison with the immobilized AgION^®^ on stainless steel, which showed a 5.6 log-reduction, but after 4 h of contact [58]. Overall, the situation is repeated when we consider the killing of *S. aureus*. Literature reports mentioned the use of TiO_2_ powders [53], immobilized/supported TiO_2_ on polymers (polystyrene, Plexiglas) [54,55] and Ag [51,57,58] systems, but also the use of negative air ions generated with the help of electrical fields [52]. None of them display comparable results in terms of efficiency or time response, this being particularly true for the immobilized TiO_2_ systems. An additional and illustrative comparison comes from the data reported by the use of different antibiotics/biocidals coating the EVOH polymer [59]. Only a powerful antibiotic, gatifloxacin, gives comparable results in terms of log-reduction, but for a certainly extended period of times (24 h), derived in the case of the antibiotic for its slow release from the polymer matrix. A recent investigation pointed out the bactericidal activities against these two microorganisms in the absence of light of titanium hydroxyl species generated by *in situ* sol-gel within polypropylene [62]. Authors postulated that these species participated to specific nanomaterial-cell interactions, and although cell death in the dark was less pronounced than in the light, titanium-based species caused oxidative stress under non-photocatalytic conditions.

**Figure 8:**
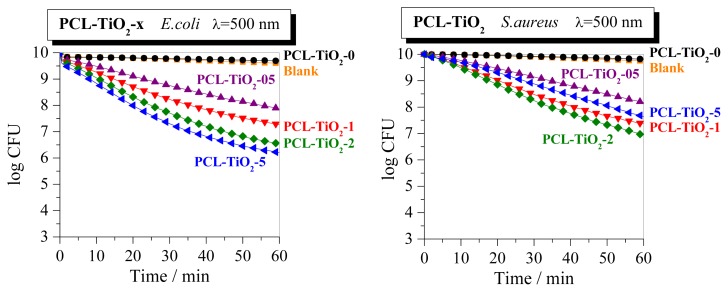
Survival curves after irradiation at 500 nm for two microorganisms (*E. coli* and *S. aureus*, top and bottom plots, respectively) as a function of the irradiation time for PCL-TiO_2_-*x* and PCL control samples.

Comparison with other systems previously prepared and analyzed by us indicates that PCL-TiO_2_-*x* nanocomposites behave under UV irradiation slightly better than those based on EVOH copolymer [17,63] and slightly worse than the ones prepared from isotactic polypropylene [11]. It should be commented that these last hybrids incorporated an interfacial agent to improve organic/inorganic contacts at interfaces, while those here analyzed and the ones prepared using EVOH as polymeric matrix are binary systems. On the other hand, the biocidal capabilities in the UV region were further improved in the EVOH based nanocomposites against *E. coli* after doping with Ag and Zn on the TiO_2_ inorganic component [17,41,63]. The purpose of doping was to be able to spread out the absorption light into the visible region of the electromagnetic spectrum, since, for instance, Ag exhibits a very intense localized surface plasmon (LSP) absorption band in the near-UV-visible region. Therefore, the potential of working under sunlight and/or diffuse artificial light, typical of human environments, without requiring UV excitation could be promoted. It was found that the antimicrobial performance of the TiO_2_-Ag films was maintained upon visible-light excitation (500 nm), although it was lowered compared with that presented by those TiO_2_-Ag films after UV irradiation. Nevertheless, this TiO_2_-Ag biocidal response was much higher than the modest performance of the EVOH/TiO_2_ nanocomposites at 500 nm [17,41,63]. These features observed in TiO_2_-Ag films were also found using Zn as dopant, while results after doping TiO_2_ with Cu [41] were similar to those displayed by EVOH/TiO_2_ nanocomposites. The biocidal behavior of those nanocomposites was associated with the presence of true, new electronic states [12,17,63], unequivocally ascribable to the nanocomposite system, as confirmed by photoluminescence. The samples here under study have been also exposed at 500 nm in order to establish a comparison with those aforementioned systems. Figure 8 shows the striking results found in these PCL-TiO_2_-*x* specimens, since they display very significant biocidal properties against both microorganisms at 500 nm. The PCL-TiO_2_-5 system is the optimum one for killing *E. coli*, whereas PCL-TiO_2_-2 exhibits the optimized response against *S. aureus*. The reason behind this extraordinary behavior upon visible-light excitation is not yet well understood, and further analyses are required for a complete comprehension.

## 3. Experimental Section

Nanocomposite preparation: The TiO_2_ component was prepared using a microemulsion synthetic route and calcined at 450 °C (characteristic primary particle size below 10 nm) [64]. These novel materials were prepared through melt processing at 140 °C and at 100 rpm for 15 min in a miniextruder Thermo Scientific Haake Minilab. Previous to this, nanoparticles and PCL were sonicated (Sonics VC505) and stirred to homogenize the batch. After their mixing, specimens were obtained as films by compression molding in a Collin press between hot plates (120 °C) at a pressure of 1.5 MPa for 5 min. A quench was applied to the different films from the melt to room temperature. The resulting nanocomposites were labeled as PCL-TiO_2_-*x*, where *x* corresponds to the weight percentage of titania content.

Nanocomposites characterization: Transmission electron microscopy (TEM) was performed at room temperature in a 200 kV JEM-2100 JEOL microscope to analyze material homogeneity. Samples were cut in thin sections (40 nm) by cryo-ultramicrotomy (Leica EM UC6). Specimens were then picked up on copper grids. Wide and small angle X-ray scattering, WAXS and SAXS, respectively, experiments employing synchrotron radiation (λ = 0.150 nm) were performed at the beamline A2 at HASYLAB (Hamburg, Germany). A MARCCD detector was used for data acquisition, sited at a distance from the sample of 25 and 265 cm, for WAXS and SAXS measurements, respectively. Calibration was performed in the WAXS region with the distinct diffractions of a triclinic semicrystalline poly(ethylene terephthalate) (PET) sample, and the SAXS one was calibrated with the different orders of the long spacing of a rat-tail cornea (λ = 65 nm). The two-dimensional X-ray patterns were processed with the A2tool program, developed to support beamline A2 data processing. The profiles were normalized to the primary beam intensity and the background from an empty sample was subtracted. All experiments comprise the heating of samples from 20 up to 76 °C at 8 °C/min. The data acquisition was done in frames of 15 s. The phase transitions were analyzed by differential scanning calorimetry measurements performed in a TA Instruments TA2000 calorimeter connected to a cooling system and calibrated with different standards. Samples (~5 mg) were scanned from −80 to 90 °C at 10 °C/min under dry nitrogen (50 cm^3^/min). The weight loss was estimated by thermogravimetry using TA Instruments TGA Q500 equipment working under inert nitrogen and air atmospheres (90 cm^3^/min). The equipment was calibrated according to standard protocols. The sample weights ranged from 4 to 6 mg, and the heating rate was 10 °C/min.

Microbiological tests: The microorganisms used in this study include *E. coli* 1337-H and *S. aureus* 1341-H and were obtained from the German Collection of Microorganisms and Cell Cultures (DSMZ, Braunschweig, Germany) and cultured and maintained according to the recommendations of the suppliers [65]. Briefly, *E. coli* 1337-H and *S. aureus* 1341-H were grown in Luria-Bertani (LB) medium at 37 °C using 100 mL flasks filled with 10 mL of the respective medium and subsequently used for photochemical cell viability assays. To study the antimicrobial activity of films, a suspension containing 10 μL of microbial cells (*ca*. 10^9^ CFU/mL) suspended in 1 mL broth solution was made [66]. Aliquots of 1 mL from these suspensions were added to a 4 mL quartz cubic cell containing 1 mL of sterilized water and the corresponding film under continuous stirring. The film-cell slurry was placed in the UV spectrometer chamber (UVIKON 930) and irradiated with a light at 280 (UV) and 500 (visible) nm for different time periods. The four nanocomposite samples (PCL-TiO_2_-*x*) together with two blanks (*i.e*., PCL + irradiation and irradiation alone) were measured using the same bacterium inoculum for each microorganism tested. As demonstrated by blank experiments, care was put of using a sub-lethal, maximum radiation energy fluence of *ca*. 1 kJ/m^2^ throughout the study. After irradiation and for different time intervals, aliquots of 100 μL were transferred to a 10 mL LB broth test tube. The order of cell dilution at this stage was 10^−2^. Loss of viability after each exposure time was determined by the viable count procedure on Luria Bertani agar plates after serial dilution (10^−2^ to 10^−5^). All plates were incubated at 37 °C for 24 h prior to enumeration. A minimum of three experimental runs were performed to determine antimicrobial activity; this leads to a standard error of ±0.1 log CFU/mL units in the reported results.

## 4. Conclusions

The characterization of PCL-TiO_2_-*x* nanocomposites with different anatase TiO_2_ contents ranging from 0 to 5 wt.% provides evidence of the nanometric dispersion of the oxide component on the polymeric matrix, with average aggregates well below 100 nm. Moreover, dependence of crystalline characteristics is subtle on anatase contents in terms of crystallinity degree, long spacing, most probable crystal size and melting temperature. Concerning the melting process, the PCL-based specimens must have an identical and well-defined thermal protocol in order to obtain reproducible and reliable results, since PCL macrochains evolve with time, leading to multiple melting stages, due to their low glass transition temperature. These multiple melting processes are ascribed to successive melting-recrystallization phenomena. Moreover, an evident nucleating effect is observed during crystallization process because of the TiO_2_ nanoparticle presence.

On the other hand, incorporation of TiO_2_ nanoparticles does not change much PCL degradability independently of the environment used, either inert or oxidant one. A good agreement is found when comparing the averaged values with the theoretical loadings from the residual mass at the end of the experiments.

The absorption profiles undergo important changes in these nanocomposites when compared with that exhibited by PCL matrix: enhancement of signal intensity in the UV-A and visible ranges and indication of the characteristic oxide band gap, this last feature only clearly distinguishable in the sample with the highest TiO_2_ content (PCL-TiO_2_-5).

The presence of TiO_2_ extremely affects the bio-killing action in the nanocomposite materials under UV irradiation. A log-reduction ranging from 3 to 5.6 is observed depending on TiO_2_ content at the end of the experiments against *E. coli*, whereas it is ranging from four to five for *S. aureus*. An evident dependence on TiO_2_ content is seen against *E. coli*, although behavior is similar for the distinct nanocomposites above 0.5 wt.% against *S. aureus*. Nevertheless, the most remarkable and extraordinary result found in these nanocomposites is their biocidal response at 500 nm against both microorganisms analyzed. The PCL-TiO_2_-5 system is the optimum one for killing *E. coli*, whereas PCL-TiO_2_-2 exhibits the optimized response against *S. aureus*.

## Figures and Tables

**Table 1:** Characteristics of the PCL crystalline phase for the different nanocomposites and for the pristine PCL homopolymer: *f_c_*^NORM^_WAXS_ (crystallinity degree determined by WAXS at room temperature); *d*^HALO^ (spacing of the amorphous halo in the molten state); *L*^SAXS^ (long spacing estimated by small angle X-ray diffraction (SAXS) at room temperature) and *l_c_* (most probable crystal size calculated assuming a two-phase model: *l*_c_ = *L*SAXS × *f_c_*^NORM^_WAXS_.

Specimen	TiO_2_ wt.%	*f_c_*^NORM^_WAXS_	*d*^HALO^ (nm)	*L*^SAXS^ (nm)	*l_c_* (nm)
PCL-TiO_2_-0	0.0	0.53	0.448	15.8	8.4
PCL-TiO_2_-05	0.5	0.54	0.448	15.8	8.5
PCL-TiO_2_-1	1.0	0.54	0.449	16.1	8.7
PCL-TiO_2_-2	2.0	0.53	0.449	16.1	8.5
PCL-TiO_2_-5	5.0	0.53	0.450	16.0	8.5

Standard errors: *fc*^NORM^_WAXS_ ± 4%; *L*^SAXS^ and *l*_c_ ± 0.3 nm.

**Table 2:** Phase transition temperatures: glass transition temperature (*T_g_*), melting temperature (*T_m_*^F1^ and *T_m_*^F2^, first and second melting processes, respectively) and crystallization temperature (*T_c_*); normalized crystallinities (first and second melting processes, as well as crystallization: *f_c_*^NORM^_F1_, *f_c_*^NORM^_F2_ and *f_c_*^NORM^_C_, respectively) a.

Specimen	*T_g_* (°C)	*f_c_*^NORM^_F1_	*T_m_*^F1^ (°C)	*f_c_*^NORM^_C_	*T_c_* (°C)	*f_c_*^NORM^_F2_	*T_m_*^F2^ (°C)
PCL-TiO_2_-0	−60.0	0.68	60.0	0.47	32.5	0.59	55.0
PCL-TiO_2_-05	−60.5	0.66	59.5	0.47	36.0	0.59	55.5
PCL-TiO_2_-1	−60.5	0.66	59.5	0.46	36.0	0.58	56.0
PCL-TiO_2_-2	−60.0	0.66	59.5	0.47	34.5	0.59	55.5
PCL-TiO_2_-5	−60.0	0.65	60.0	0.46	34.0	0.58	55.5

a Estimated errors: temperatures ± 0.5 °C; crystallinity ± 5%.
